# Infectious complications of radiologically placed upper arm ports: A single center analysis

**DOI:** 10.1371/journal.pone.0284475

**Published:** 2023-04-13

**Authors:** Daniel Koehler, Jan-Marcus Haus, Farzad Shenas, Holger Rohde, Harald Ittrich, Gerhard Adam, Kersten Peldschus

**Affiliations:** 1 Department of Diagnostic and Interventional Radiology and Nuclear Medicine, University Medical Center Hamburg-Eppendorf, Hamburg, Germany; 2 Institute of Medical Microbiology, Virology and Hygiene, University Medical Center Hamburg-Eppendorf, Hamburg, Germany; Shiraz University of Medical Sciences, ISLAMIC REPUBLIC OF IRAN

## Abstract

**Objectives:**

Infections are common complications in venous access ports. The presented analysis aimed to investigate the incidence, microbiological spectrum, and acquired resistances of pathogens in upper arm port associated infections to provide a decision aid in the choice of therapy.

**Materials and methods:**

In total, 2667 implantations and 608 explantations were performed at a high-volume tertiary medical center between 2015 and 2019. In cases with infectious complications (n = 131, 4.9%), procedural conditions and results of microbiological testing were reviewed retrospectively.

**Results:**

Of 131 port associated infections (median dwell time 103 days, interquartile range 41–260), 49 (37.4%) were port pocket infections (PPI) and 82 (62.6%) were catheter infections (CI). Infectious complications occurred more often after implantation in inpatients compared to outpatients (P < 0.01). PPI were mainly caused by *Staphylococcus aureus* (*S*. *aureus*, 48.3%) and coagulase-negative staphylococci (CoNS, 31.0%). Other gram-positive and gram-negative species were encountered in 13.8% and 6.9%, respectively. CI were caused less frequently by *S*. *aureus* (8.6%) than CoNS (39.7%). Other gram-positive and gram-negative strains were isolated in 8.6% and 31.0%, respectively. *Candida* species were seen in 12.1% of CI. An acquired antibiotic resistance was detected in 36.0% of all significant isolates, occurring especially in CoNS (68.3%) and gram-negative species (24.0%).

**Conclusions:**

Staphylococci comprised the largest group of pathogens in upper arm port associated infections. However, gram-negative strains and *Candida* species should also be considered as a cause of infection in CI. Due to the frequent detection of potential biofilm-forming pathogens, port explantation is an important therapeutic measure, especially in severely ill patients. Acquired resistances must be anticipated when choosing an empiric antibiotic treatment.

## Introduction

Long-lasting intravenous therapies (e.g. chemotherapy in malignancies, long term antibiotic courses in chronic infections, nutrition, etc.) together with repeated blood sampling are extremely strenuous for chronically ill patients. To improve the quality of life in these individuals and to create a safe venous access, totally implantable venous access ports (short: ports) were introduced in 1982 [1]. The most common site for the implantation of a port has been the chest wall. However, upper arm ports were established as a feasible alternative [2]. Comparable to other venous access devices, infections are a common complication of arm ports with a frequency of 2.0% - 9.9% [3–10]. Port associated infectious complications are subdivided into local infections of the port pocket (PPI) and catheter infections (CI). PPI must be considered when swelling, erythematic induration, pain, and/or pus are encountered at the port site. CI may be assumed when blood culture tests or signs of systemic inflammation (i.e. elevated C-reactive protein, white blood cell count, body temperature) are positive and no other focus can be found [4]. Infections pose a threat, especially to immunocompromised patients, causing increased morbidity and mortality [11,12]. Therefore, an empiric antimicrobial therapy is usually initiated after specimens for microbiological testing have been obtained. The choice of an empiric antibiotic treatment is of high importance and may be complicated by changes in the bacterial spectrum or evolving drug resistances. Knowledge of the local epidemiology of bacterial strains should comprise the basis of treatment [11]. Information on the microbiological spectrum in arm port infections is scarce. *Staphylococcus aureus (S*. *aureus)*, *Staphylococcus epidermidis (S*. *epidermidis)*, *Enterobacter cloacae (E*. *cloacae)*, and *Candida* species (spp.) were reported, but susceptibility profiles of isolated pathogens are lacking [5,7].

The aim of this study was to analyze the incidence, microbiological spectrum, and acquired resistances of pathogens in the setting of upper arm port associated infections to provide a decision aid in the choice of therapy in these cases.

## Materials and methods

### Study cohort

Between January 1^st^ 2015 and December 31^st^ 2019, a total of 2667 upper arm port implantations and 608 explantations were performed in the interventional radiology department of a tertiary referral hospital. All patients and/or their legal guardian(s) provided their written informed consent for the procedures. The local institutional review board (ethics committee of the medical council of the Free and Hanseatic City of Hamburg) waived the requirement for informed consent regarding the retrospective analysis of the derived data. All scientific methods were conducted in accordance with the Declaration of Helsinki.

**Fig 1:**
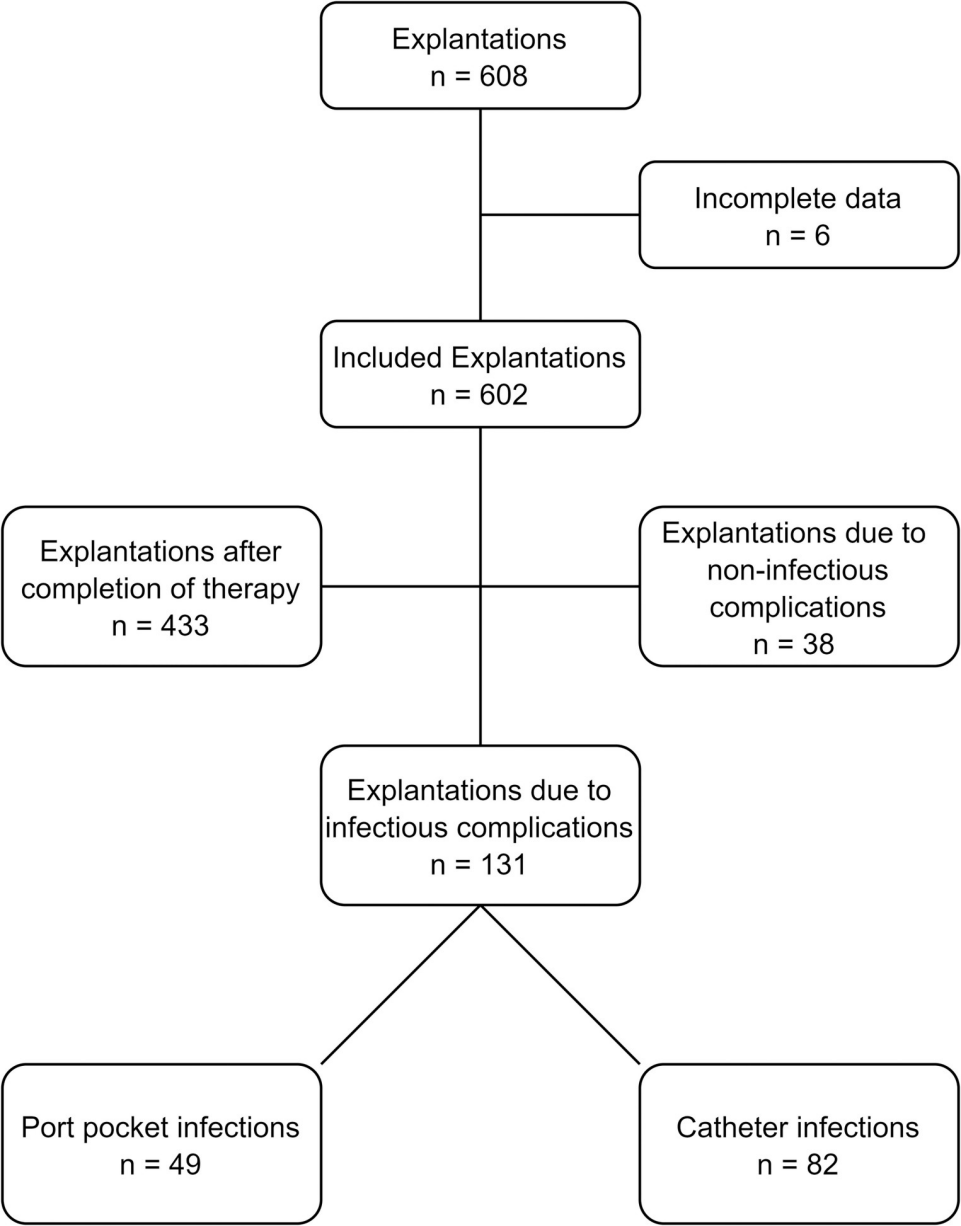
Case selection flow chart.

Six cases were excluded from the further analysis of explanted ports due to incomplete data (Fig 1).

### Surgical technique and post-operative care

**Fig 2:**
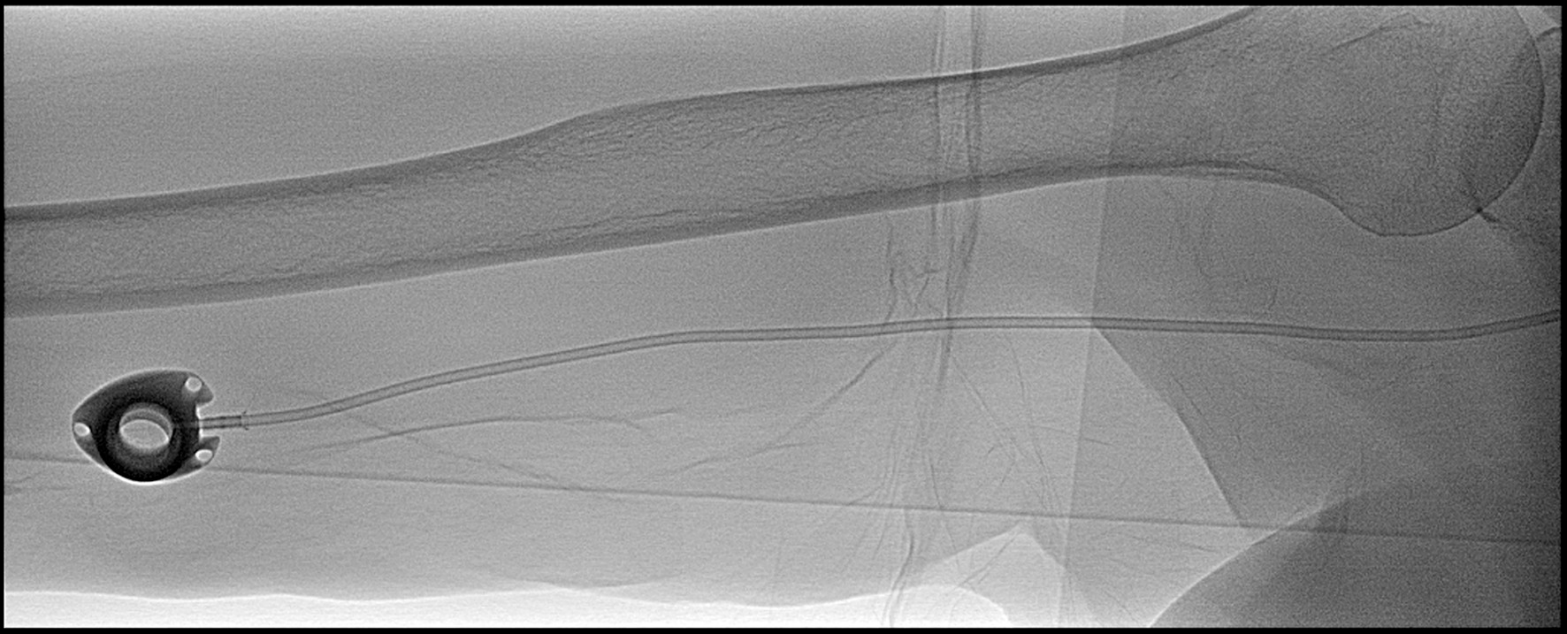
Bard Titanium SlimPort^TM^. Fluoroscopic image of a Bard Titanium SlimPortTM with a 6F single lumen polyurethane catheter in typical location at the distal upper arm.

All ports were Bard Titanium SlimPorts^TM^ with a 6F single lumen polyurethane catheter (Bard Access Systems, Inc., Salt Lake City, Utah, USA; Fig 2). The recommended maximum number of punctures of the port membrane was 1000 times with a 22-gauge needle or 500 punctures with a 19-gauge needle. Port implantations were conducted in local anesthesia under aseptic conditions by specially trained radiologists in a designated interventional radiology suite. The institutional standard regarding the period between implantation and first use of a port changed during the study period. Until February 2017, at least 24h were kept between the implantation and first utilization, affecting 345 of the analyzed explantations. In the following 257 instances, ports had been used from the day of implantation. Surgical sites were inspected either by radiologists or by the treating physicians 24 – 48h after implantation. Afterwards, wound dressings were changed regularly by medical personnel until wound closure. Sutures were removed after 8–10 days. All medical personnel who were involved in the care of patients with upper arm ports were trained in their use and maintenance. Yearly training in hospital hygiene, including hand hygiene and hygienic application of intravenously administered substances, was mandatory for all medical staff involved in direct patient care at the institution. Insertion of port needles was conducted under aseptic conditions. Port sites were checked regularly for signs of infection or disconnection during use. After the insertion of the port system and every time before the port needle was removed, the port was flushed with 500 international units of heparin in 10 ml of 0.9% sodium chloride solution. No antibiotic lock prophylaxis or antimicrobial catheter flush were installed in the study cohort.

### Definition of port associated infections and interpretation of microbiological findings

Port associated infectious complications were subdivided into PPI and CI.

PPI (Group 1) were considered if the port site was reddened, swollen, painful, and/or pus was encountered at the insertion site or the injection site. If fever and/or leukocytosis were present without another source of inflammation, a PPI associated systemic infection was assumed. Catheter tips were routinely analyzed in PPI. Microbiological testing of blood cultures was mandatory in patients with PPI and an associated systemic infection. All recovered isolates from catheter tips of patients with PPI were seen as significant. In cases of PPI with associated systemic infections, results of the microbiological testing were only considered if the recovered strains from blood cultures and the corresponding catheter tip were identical.

A CI (Group 2) was assumed in patients with signs of a systemic inflammation (at least one of the following: fever, leukocytosis, elevated c-reactive protein and/or elevated procalcitonin) but without a PPI or any other apparent source of infection. Results of the catheter tip analysis were included if available. Blood cultures were routinely analyzed in patients with CI.

Isolates from blood cultures were interpreted as significant in CI if they matched the species recovered from the corresponding catheter tip (Group 2.1). Alternatively, in cases with a negative result of the catheter tip, blood cultures were interpreted as significant if the same species was recovered from more than one blood culture or the time to positivity was <24h in bacterial isolates (Group 2.2). All CI with evidence of fungi in a blood culture were included.

Port explantations due to infectious complications were categorized into periprocedural (<24 hours), early (≤30 days) and late (>30 days) events as described elsewhere [13–15].

### Microbiological specimen processing

Blood cultures (BD Bactec Plus aerobic and anaerobic medium, BD, Heidelberg, Germany) were incubated in BactecFx instruments for five days.

Samples flagged positive were subjected to Gram-stain and sub-cultured on Columbia blood agar (Oxoid, Bremen, Germany). If fungi became evident, blood cultures were additionally streaked onto Sabouraud agar. Bacteria and fungi were differentiated to the species level by whole cell mass spectrometry (MALDI Biotyper, Bruker, Bremen Germany) and susceptibility was tested using Vitek2 instruments (Biomerieux, Marcy L´Étolie, France). Catheter tips were analyzed using the roll plate technique. After overnight incubation colonies were counted.

Results of antibiotic susceptibility testing were reviewed for the included isolates from catheter tip cultures and blood cultures of PPI and CI. If isolates of the same species with identical resistance patterns were seen in more than one specimen from one patient (i.e. multiple blood cultures), they were regarded as clonal and all but one isolate were removed from the analysis. A resistance phenotype was defined as acquired (i.e. not intrinsic) if wild type minimum inhibitory concentrations of a given species against a given antimicrobial agent fell into the “susceptible” category according to the European Committee on Antimicrobial Susceptibility Testing breakpoints [16].

### Statistical analysis

Analyses were conducted using Excel 2016 Professional (Microsoft Corporation, Redmond, Washington) and GraphPad Prism 6 (GraphPad Software, San Diego, USA). Continuous variables were described by means and standard deviation (SD) or median and interquartile range (IQR). Chi-Square test with Yate’s correction or Fisher’s exact test were used for categorical data. A *P*-value of ≤ 0.05 was considered significant.

## Results

The total dwell time (DT) of the reviewed 602 arm ports was 210775 days (d) with a mean DT of 350.1d (SD 368.3d). 582 (96.7%) ports had been implanted for chemotherapy and 20 (3.3%) for repetitive intravenous therapy or nutrition. 433 arm ports (71.9%) were removed after completion of therapy (DT: median 245d, IQR 174.5 - 450d), 131 (21.8%) due to infectious complications (DT: median 103d, IQR 41 - 260d), and 38 (6.3%) due to non-infectious complications (DT: median 164.5d, IQR 24.5–881.5d). Non-infectious complications included the irreversible thrombosis of the port catheter (n = 2, 0.3%), irritation of arm nerves after port placement (n = 4, 0.7%), dysfunction or malpositioning of the port system (n = 14, 2.3%), and the irreversible thrombosis of the catheter vein (n = 18, 3.0%). Main case demographics are summarized in Table 1.

**Table 1:** Case characteristics of all upper arm port explantations and explantations due to infectious complications.

	All explantations (n = 602)	Explantation due to infections (n = 131)
Sex		
Female	409 (67.9%)	67 (51.1%)
Male	193 (32.1%)	64 (48.9%)
Age in years (mean ± SD)	55 ± 14	59 ± 14
Side		
Left	431 (71.6%)	92 (70.2%)
Right	171 (28.4%)	39 (29.8%)
Catheter days (median, IQR)	223d, 139.8–413.5d	103d, 41 - 260d
Underlying disease		
Breast cancer	211 (35%)	13 (9.9%)
Lung cancer	20 (3.3%)	6 (4.6%)
Head and neck cancer	104 (17.3%)	14 (10.7%)
Gastrointestinal cancer	52 (8.6%)	31 (23.7%)
Gynecological cancer	59 (9.8%)	18 (13.7%)
Lymphoma/leukemia	103 (17.1%)	32 (24.4%)
Urogenital cancer	15 (2.5%)	3 (2.3%)
Other malignancies	18 (3%)	7 (5.3%)
Non-malignant disease	20 (3.3%)	7 (5.3%)

SD–standard deviation, IQR–interquartile range.

### Infectious complications

A total of 131 upper arm ports were explanted due to infectious complications, i.e. 4.9% of 2667 implantations during the study period. 37.4% (n = 49) presented with PPI, including 13 (26.5%) cases with local inflammation and a systemic infection. CI were diagnosed in 62.6% (n = 82) of patients (Table 2).

**Table 2:** Case characteristics of port pocket infections (PPI) and catheter infections (CI).

	PPI (n = 49)	CI (n = 82)
Sex		
Female	24 (49%)	43 (52.4%)
Male	25 (51%)	39 (47.6%)
Age in years (mean ± SD)	59 ± 14	59 ± 14
Side		
Left	33 (67.3%)	59 (72%)
Right	16 (32.7%)	23 (28%)
Catheter days (median, IQR)	63, 21–167.5	114, 58–333.5
Underlying disease		
Breast cancer	10 (20.4%)	3 (3.7%)
Lung cancer	1 (2%)	5 (6.1%)
Head and neck cancer	5 (10.2%)	9 (11%)
Gastrointestinal cancer	12 (24.5%)	19 (23.2%)
Gynecological cancer	6 (12.2%)	12 (14.6%)
Lymphoma/leukemia	9 (18.4%)	23 (28%)
Urogenital cancer	1 (2%)	2 (2.4%)
Other malignancies	3 (6.1%)	4 (4.9%)
Non-malignant disease	2 (4.1%)	5 (6.1%)

PPI–port pocket infection, CI–catheter infection, SD = standard deviation, IQR–interquartile range.

No periprocedural infections were encountered, 17.6% (n = 23) of all infections occurred early (≤30d), and 82.4% (n = 108) late after implantation (>30d). The ratio of PPI to CI differed between early (PPI 69.6% vs. CI 30.4%) and late infections (PPI 30.6% vs. CI 69.4%). A period of at least 24 hours between port implantation and first use did not show a significant difference in the number of explantations due to early PPI (*P* > 0.99, odds ratio 0.9, 95% confidence interval 0.3–3.2). The number of port associated infections after an implantation in inpatients was significantly higher compared to outpatients (*P* < 0.01, odds ratio 0.4, 95% confidence interval 0.3–0.6). This was seen in CI (*P* < 0.01, odds ratio 0.4, 95% confidence interval 0.2–0.6) but not in PPI (P = 0.08, odds ratio 0.6, 95% confidence interval 0.3–1).

### Microbial species distributions in PPI and CI

Of 49 PPI, results of the catheter tip analysis were available in 45 cases (91.8%) and species were recovered in 28 cases (62.2%). Local inflammation with signs of a systemic infection was diagnosed in 13 patients (26.5%) of which 12 (92.3%) had positive blood cultures. Results of microbiological testing of the catheter tips were available in 11 cases (84.6%) of which 7 (63.6%) were positive. Of 82 CI, blood culture results were available in 81 cases (98.8%) with 66 (81.5%) positive specimens. Results of the catheter tip analysis were available in 72 cases (87.8%), 38 (52.8%) were positive.

**Fig 3:**
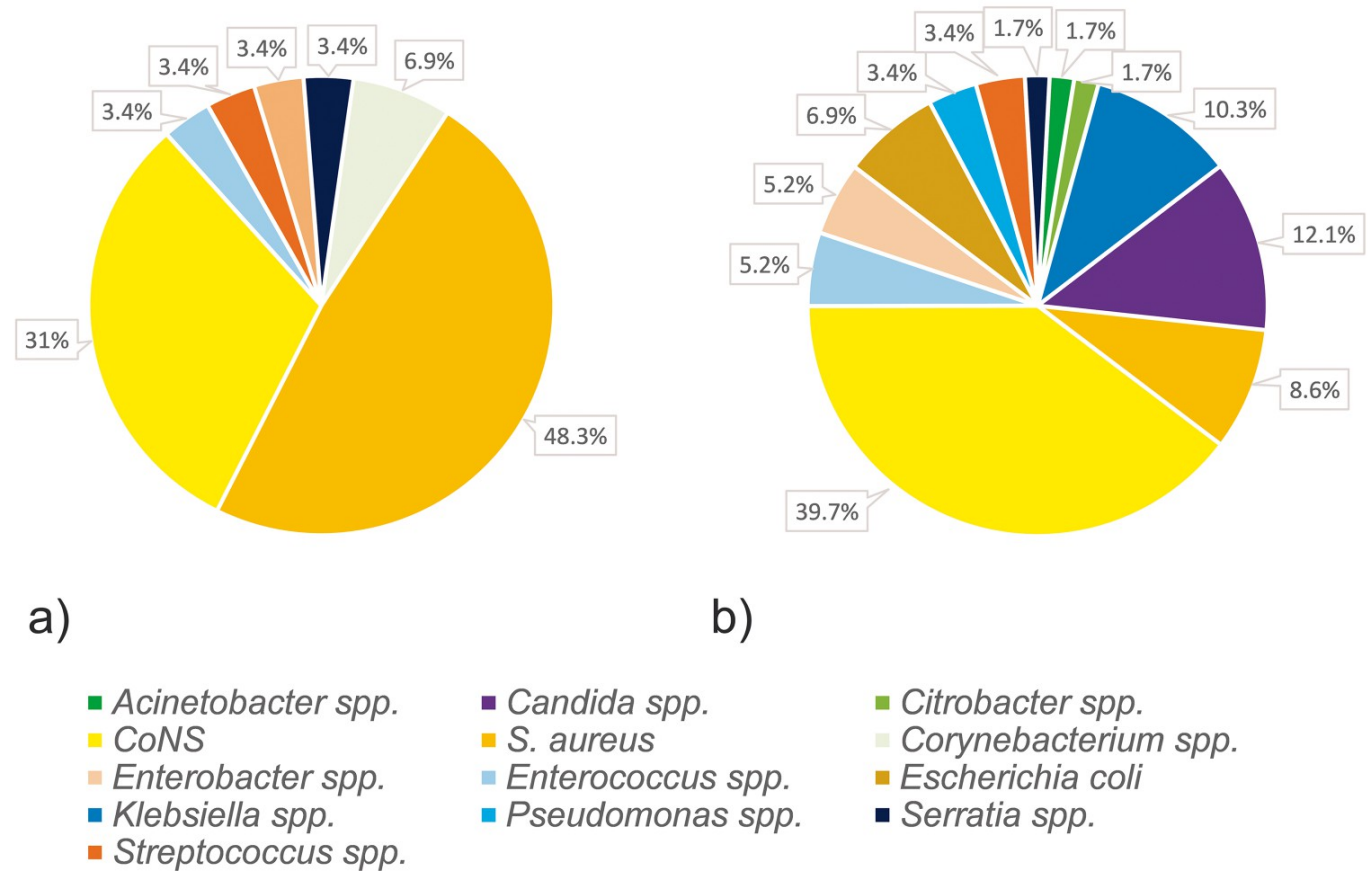
Microbiological spectrum of upper arm port associated infections. Microbiological spectrum of port pocket infections (**a**, Group 1) and catheter infections (**b**, Group 2). Coagulase-negative staphylococci–CoNS, species–spp.

A total of 160 isolates were recovered from blood cultures and 76 from catheter tips. Fig 3 shows pie charts of the microbiological spectra of significant isolates that were recovered from PPI (Group 1) and CI (Group 2) which comprised of 29 and 58 differentiated strains, respectively.

PPI were most often associated with staphylococci (79.3%) corresponding to 48.3% *S*. *aureus* and 31% coagulase-negative staphylococci (CoNS). CoNS were comprised of *S*. *epidermidis* (77.8%), *Staphylococcus haemolyticus* (*S*. *haemolyticus*, 11.1%), and *Staphylococcus caprae* (11.1%). Further gram-positive pathogens were *Enterococcus faecalis* (*E*. *faecalis*), *Streptococcus agalactiae*, *Corynebacterium amycolatum*, and *Corynebacterium aurimucosum* (3.4%, respectively). Gram-negative rods were *Serratia marcescens* (*S*. *marcescens*) and *E*. *cloacae* complex (3.4%, respectively).

Staphylococci comprised 48.3% of all pathogens in CI (*S*. *aureus* 8.6%, CoNS 39.7%). *S*. *epidermidis* accounted for 69.6% of all CoNS, followed by *Staphylococcus hominis* and *S*. *haemolyticus* (8.7%, respectively), *Staphylococcus lugdunensis*, *Staphylococcus schleiferi*, and *Staphylococcus warneri* (4.3%, respectively). Other gram-positive species (*Enterococcus faecium* [*E*. *faecium*], *E*. *faecalis*, *Streptococcus mitis* group, *Streptococcus anginosus*) were identified in five cases (8.6%). Gram-negative bacteria were detected in 31.0% of CI. *Klebsiella oxytoca* and *Klebsiella variicola* accounted for 33.3%, *Escherichia coli* for 22.2%, *E*. *cloacae* complex for 16.7%, *Pseudomonas aeruginosa* and *Pseudomonas monteilii* for 11.1%, *Acinetobacter baumannii* complex, *Citrobacter freundii* as well as *S*. *marcescens* each for 5.6%. In 12.1% of all CI, *Candida* spp. were identified.

**Fig 4:**
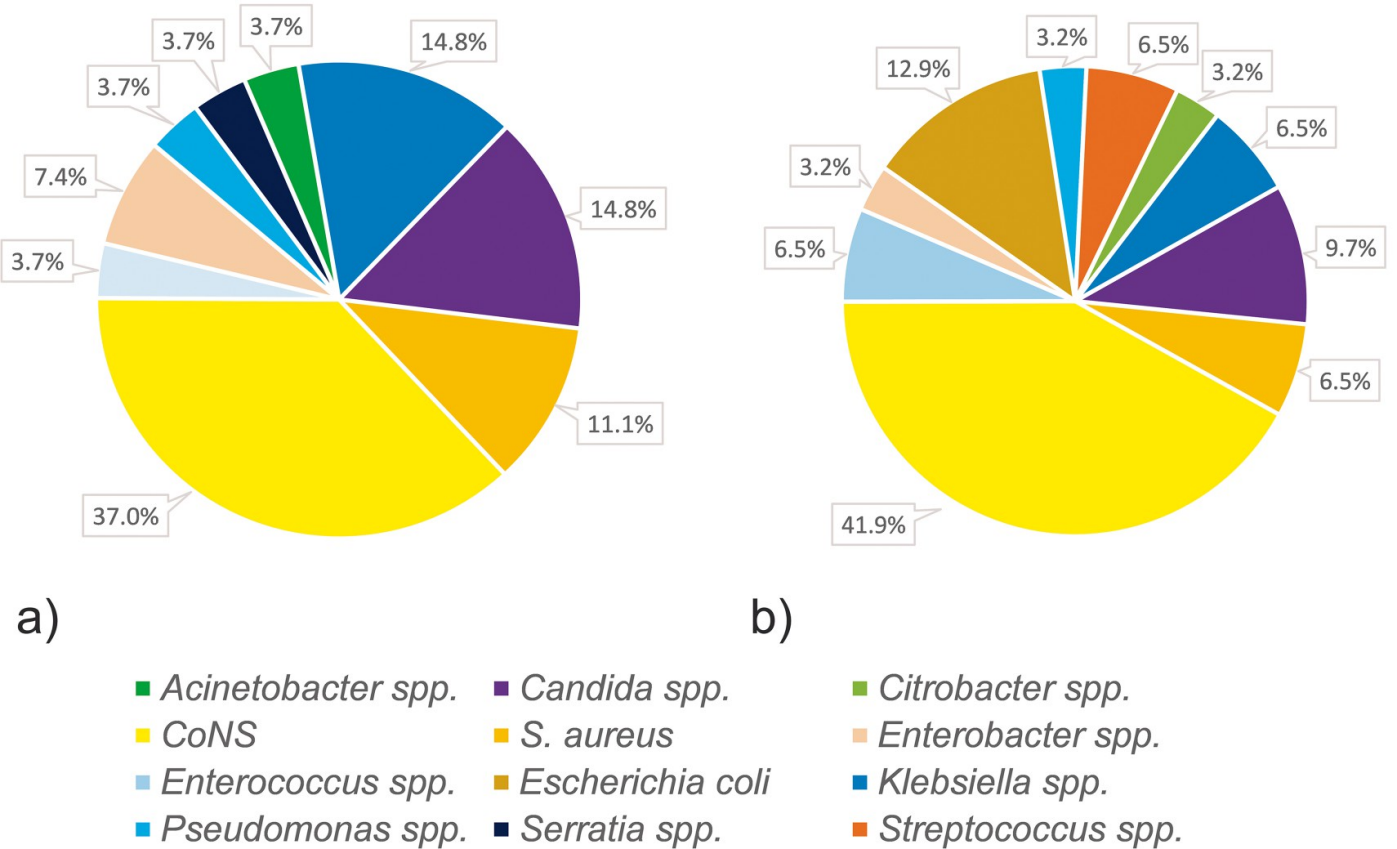
Subgroup analysis of catheter infections (CI). Group 2.1: CI with matching isolates from blood cultures and the port catheter tip (a). Group 2.2: CI with positive blood culture results and negative culture of the port catheter tip (b). Coagulase-negative staphylococci–CoNS, species–spp.

The results of the CI subgroups (Group 2.1 and Group 2.2) are summarized in Fig 4. Staphylococci were found in 48.1% (Group 2.1) and 48.4% (Group 2.2), respectively. CoNS were seen more often than *S*. *aureus* in both groups (Group 2.1: 37.0% vs. 11.1%; Group 2.2: 41.9% vs. 6.5%). The remaining isolates showed a similar variety of gram-negative species (33.3% and 29.0%, respectively) and *Candida* spp. (14.8% and 9.7%, respectively).

### Susceptibility testing

One hundred significant isolates with distinct susceptibility tests were retrieved from blood cultures and catheter tips (Table 3). Testing was not available in 4 isolates. At least one acquired resistance was found in 36.0% of all differentiated strains.

Of all CoNS, 68.3% possessed acquired resistances. All of these strains were not susceptible to methicillin. Only four CoNS (9.8%) demonstrated further resistances. These isolates were resistant to rifampicin (three from PPI, one from CI), including one strain from a PPI that was also not susceptible to linezolid in addition to methicillin and rifampicin. One isolate of *E*. *faecium* from a blood culture of a patient with a CI was not susceptible to vancomycin.

**Table 3:** Isolates with distinct susceptibility tests and frequency of strains with acquired resistances in port pocket infections and in catheter infections.

Species	All n (AR%)	PPI Group 1 n (AR%)	CI Group 2 n (AR%)	Group 2.1 n (AR%)	Group 2.2 n (AR%)
**Gram-positive bacteria**					
*Corynebacterium* spp.	1 (100%)	1 (100%)	/	/	/
*Enterococcus* spp.	4 (25%)	1 (0%)	3 (33.3%)	1 (0%)	2 (50%)
*S*. *aureus*	20 (0%)	14 (0%)	6 (0%)	4 (0%)	2 (0%)
CoNS	41 (68.3%)	8 (75%)	33 (66.7%)	16 (81.3%)	17 (52.9%)
*Streptococcus* spp.	3 (0%)	1 (0%)	2 (0%)	/	2 (0%)
**Gram-negative bacteria**					
*Acinetobacter* spp.	1 (0%)	/	1 (0%)	1 (0%)	/
*Citrobacter* spp.	1 (0%)	/	1 (0%)	/	1 (0%)
*Enterobacter* spp.	5 (40%)	1 (0%)	4 (50%)	3 (66.7%)	1 (0%)
*Escherichia* spp.	5 (60%)	/	5 (60%)	/	5 (60%)
*Klebsiella* spp.	8 (0%)	/	8 (0%)	6 (0%)	2 (0%)
*Pseudomonas* spp.	3 (33.3%)	/	3 (33.3%)	2 (50%)	1 (0%)
*Serratia* spp.	2 (0%)	1 (0%)	1 (0%)	1 (0%)	/
**Fungi**					
*Candida* spp.	6 (0%)	/	6 (0%)	4 (0%)	2 (0%)
	100 (36%)	27 (25.9%)	73 (39.7%)	38 (42.1%)	35 (37.1%)

Number of isolates (n) and frequency of strains with acquired resistances (AR%) in port pocket infections (PPI, Group1) and catheter infections (CI, Group 2). Group 2.1: CI with matching isolates from blood cultures and the port catheter tip. Group 2.2: CI with positive blood culture results and negative culture of the port catheter tip. Coagulase-negative staphylococci–CoNS, species–spp.

None of the isolated gram-negative strains from PPI displayed acquired resistances. Of 23 distinct gram-negative isolates from CI, 26.1% possessed at least one acquired resistance which corresponds to 24% of all analyzed gram-negative strains. This included strains that were non-susceptible to fluoroquinolone (e.g. ciprofloxacin, 4 isolates) and β-lactam antibiotics (e.g. 3^rd^ generation cephalosporines, piperacillin/tazobactam, 3 isolates). Results of susceptibility testing were comparable in both CI subgroups.

## Discussion

In this 5-year retrospective analysis, upper arm port associated infections were found in 131 cases corresponding to an infection rate of 4.9%. Staphylococci comprised the largest group of pathogens in port pocket infections and catheter infections. Gram-negative strains and *Candida* spp. were an important differential in catheter infections. Acquired resistances against antibiotics were seen especially in coagulase-negative staphylococci (e.g. *S*. *epidermidis*, *S*. *haemolyticus*, etc.).

The infection rate in the presented cohort is comparable to previously published data [3–10]. PPI comprised 37.4% and CI 62.6%, which is similar to the ratio reported by Mori et al. [4]. Infectious complications occurred significantly more often after an implantation in an inpatient setting compared to an outpatient scenario. Looking at the two groups, this was also the case in CI but not in PPI. Therefore, the increased infection rate in inpatients may be seen as a denominator for the severity of the underlying disease rather than procedural deficits of the port implantation. Further on, no periprocedural infections and a lower ratio of early to late infections (17.6% early vs. 82.4% late) were encountered in comparison to other publications [3,5,7]. Most early infectious complications were PPI (69.6%). An interval of at least 24 hours before first use after insertion was not associated with a reduction of early PPI (*P* > 0.99). A longer interval as suggested by Kakkos et al. in chest ports [17] could be beneficial. But since the early complication rate was already relatively low, a further reduction may be difficult to achieve.

Only few reports on the microbiological spectrum of arm port associated infections are available. Mori et al. stated that bacteria and fungi were isolated [4]. Bodner et al. described positive blood cultures in two patients with PPI (*S*. *aureus* & *S*. *epidermidis*, respectively) and in two cases of CI (both *S*. *epidermidis*) [7]. Busch et al. reported that PPI were caused by *S*. *aureus* as well as *Candida* spp. In septicemia, methicillin-resistant *S*. *aureus* (MRSA) and *E*. *cloacae* were detected in blood cultures [5]. In the presented study, the microbiological spectrum of PPI was mainly comprised of gram-positive species, dominated by *S*. *aureus* and CoNS (*S*. *aureus* 48.3%, CoNS 31%). Gram-negative species were only found sporadically, and fungi were not detected at all.

Though less frequent than in PPI, staphylococci were responsible for almost half of all CI (48.3%) with CoNS causing substantially more infections than *S*. *aureus* (39.7% vs. 8.6%). The remaining microbiological spectrum in CI was more diverse than in local infections. A variety of gram-negative strains or *Candida* spp. were encountered. The subgroup analysis of CIs showed similar microbiological spectra regardless of the results of the catheter tip. Therefore, the analysis of the port catheter tip may confirm the diagnosis of a CI but it often does not provide additional information. Moreover, it should be noted that the method is prone to contamination. In patients with laboratory proof of a bloodstream infection, a standard analysis of the catheter may hence not be helpful. However, microbiological testing of the port catheter tip should be considered in inconclusive cases with persistent signs of infection.

Acquired resistances of pathogens against antimicrobial agents are a major health threat [18–20]. Comparable to a recent review [21], 68.3% of all isolated CoNS were not susceptible to methicillin. Only 9.8% of strains possessed further acquired immunities, including a non-susceptibility against oxazolidinones (e.g. linezolid) in one case. In contrast, none of the analyzed *S*. *aureus* strains was resistant to methicillin which corresponds to the reported decrease of MRSA infections in the past years [22,23]. 24% of all gram-negative strains demonstrated acquired resistances. These were mostly against fluoroquinolone (e.g. ciprofloxacin) and β-lactam antibiotics (e.g. piperacillin/tazobactam). *Candida* spp. had no acquired resistances.

Potential biofilm-forming pathogens (e.g. staphylococci, *Klebsiella* spp., *Candida* spp., etc.) were found frequently in the investigated cohort. Therefore, port explantation is seen as an important measure, especially in severely ill patients. An antibiotic treatment should be adjusted according to the clinical presentation of each case considering the vulnerability of cancer patients. In the presented cohort, *S*. *aureus* was the primary pathogen in PPI followed by CoNS. An appropriate empiric antibiotic treatment after explantation could be an oral antimicrobial agent with high activity against gram-positive bacteria (e.g. cephalosporines of the 1^st^ generation like cefalexin) if no signs of a systemic inflammation are evident. In PPI with a systemic infection and in CI, an intravenous administration of vancomycin could be reasonable due to the high rate of infections caused by staphylococci, including CoNS which were frequently resistant to methicillin. Furthermore, the addition of an antibiotic against gram-negative strains may be beneficial in the empiric treatment of CI (e.g. meropenem).

Limitations of this study include its retrospective design which may have led to an underestimation of infectious complications due to incomplete reporting and a selection bias in our patient collective. Moreover, the presented data were collected at a single institution representing only the spectrum at this hospital. Also, the techniques for pathogen differentiation as well as susceptibility testing may differ from other centers, limiting the comparison of results. The investigation at a single tertiary center may have resulted in a selection bias. Due to the mostly severe course of the primary disease in the investigated patient cohort, port explantations were routinely conducted. Further analyses at multiple centers are needed to investigate if microbiological spectra differ between institutions or regions and if different therapeutic approaches are eligible in arm port associated infections.

### Conclusions

Upper arm port associated infections are a common complication. Staphylococci comprised the largest group of pathogens both in PPI and CI. However, gram-negative strains and *Candida* species should also be considered as a possible cause of infection in CI. Due to the frequent detection of potential biofilm-forming pathogens, port explantation is an important therapeutic measure, especially in severely ill patients. Acquired antibiotic resistances were frequent in certain pathogen subgroups, e.g. CoNS and gram-negative strains, and should be anticipated when choosing an empiric antibiotic treatment.

## Supporting information

S1 File Case related data of port explantations. (XLSX)
